# Targeted inhibition of GRP78 by HA15 promotes apoptosis of lung cancer cells accompanied by ER stress and autophagy

**DOI:** 10.1242/bio.053298

**Published:** 2020-11-12

**Authors:** Jingjing Wu, Youqile Wu, Xuemei Lian

**Affiliations:** 1 Center for Lipid Research, Key Laboratory of Molecular Biology for Infectious Diseases (Ministry of Education), Chongqing Medical University, Chongqing 400016, P.R. China; 2 Department of Nutrition and Food Hygiene, School of Public Health and Management, Chongqing Medical University, Chongqing 400016, P.R. China; 3 Department of Child Health Care, Mianyang Maternity and Child Healthcare Hospital, Sichuan 621000, P.R. China

**Keywords:** GRP78, Lung cancer, ER stress, Autophagy

## Abstract

This study investigated the pathophysiological role of GRP78 in the survival of lung cancer cells. Lung cancer patient data from public databases were used to analyze the expression of GRP78 and its influence on prognoses. *In vivo*, GRP78 protein expression was analyzed in an established urethane-induced lung tumor mouse model. *In vitro*, the effects of targeted inhibition of GRP78 by HA15 in lung cancer cells were assessed, with cell viability analyzed using a CCK-8 assay, cell proliferation using an EdU assay, apoptosis and cell cycle using flow cytometry, subcellular structure using electron microscopy, and relative mRNA and protein expression using RT-PCR, western blotting or immunofluorescence assays. The results showed that GRP78 was highly expressed in the lung tissue of lung cancer mice model or patients, and was associated with a poor prognosis. After inhibition of GRP78 in lung cancer cells by HA15, cell viability was decreased in a dose- and time-dependent manner, proliferation was suppressed and apoptosis promoted. Unfolded protein response signaling pathway proteins were activated, and the autophagy-related proteins and mRNAs were upregulated. Therefore, targeted inhibition of GRP78 by HA15 promotes apoptosis of lung cancer cells accompanied by ER stress and autophagy.

## Abstract (precis)

**Summary:** GRP78 is highly expressed in the lung cancer tissue, and targeted inhibition of GRP78 by HA15 promotes apoptosis of lung cancer cells accompanied by endoplasmic reticulum stress and autophagy.

## INTRODUCTION

Lung cancer is the most frequently diagnosed cancer and the most common cause of cancer-related deaths worldwide. Deaths caused by lung cancer are more common than breast, prostate, colorectal and brain cancers combined, causing a huge public health and financial burden (Siegel et al., 2020). Generally, the current therapies for lung cancer include surgery, chemotherapy, and radiotherapy. Due to late diagnosis and drug resistance, the 5-year survival rate of lung cancer is approximately only 19% (Siegel et al., 2020).

Glucose-regulated protein 78 (GRP78) also known as biding-immunoglobulin protein (BiP) or heat shock protein family A (HSP70) Member 5 (HSPA5), is located on the endoplasmic reticulum (ER) membrane in all eukaryotes, where it plays a crucial role in protein folding and maturation (Brocchieri et al., 2008). ER stress is generally activated in cancer cells due to an accumulation of misfolded proteins occurring due to genetic mutations and genomic rearrangements. The unfolded protein response (UPR) then triggers the expression of chaperone proteins, such as GRP78, calreticulin and calnexin, which play a role in the adaption of the cell to ER stress (Dong et al., 2008). As the main ER chaperone, GRP78 is reported to promote survival and chemoresistance in tumor cells by correcting misfolded proteins and promoting recovery from ER stress (Lee, 2001, Li and Lee, 2006). However, the role of GRP78 in lung cancer is still controversial. The majority of studies reported that GRP78 is highly expressed in lung cancer patients and is associated with poor prognostic outcomes (Kwon et al., 2018; Wang et al., 2005). However Uramoto et al. hold opposing opinions (Uramoto et al., 2005).

Autophagy is a crucial process for maintaining homeostasis in the human body, eliminating damaged macromolecules or organelles (Kroemer et al., 2010). Furthermore, autophagy can protect cells from stressors and help cells overcome the harsh conditions produced by the tumor microenvironment, such as ischemia and hypoxia (Poillet-Perez and White, 2019). In primary lung tumors, autophagy associated with drug resistance has been reported as a biomarker for subclassification, differentiation, and local metastasis (Yuan et al., 2017; Chen et al., 2018). Inhibition of autophagy may be a novel clinical treatment strategy to surmount the antagonistic effects between the epidermal growth factor receptor (EGFR) tyrosine kinase inhibitor (TKI) (EGFR-TKIs) and chemotherapeutic agents (Liu et al., 2015).

HA15, the leading compound of the thiazole benzenesulfonamide, is a novel inhibitor targeting GRP78. HA15 binds to GRP78, and inhibits its ATPase activity (Cerezo et al., 2016). It has been reported that HA15 played an anti-tumor role in melanoma, breast, pancreatic and adrenocortical carcinoma, even overcoming drug resistance (Cerezo et al., 2016; Ruggiero et al., 2018). However, the effect of HA15 in lung cancer remains unclear. In this study, we found that targeted inhibition of GRP78 by HA15 induced apoptosis in lung cancer cell lines, triggering ER stress and autophagy. Our data suggest that GRP78 plays a pro-survival role in human lung cancer cells and is related to poor prognosis; inhibition of GRP78 by HA15 might be effective as a novel therapy in clinical settings.

## RESULTS

### GRP78 is highly expressed in lung cancer and is associated with poor prognoses

**Fig. 1.**
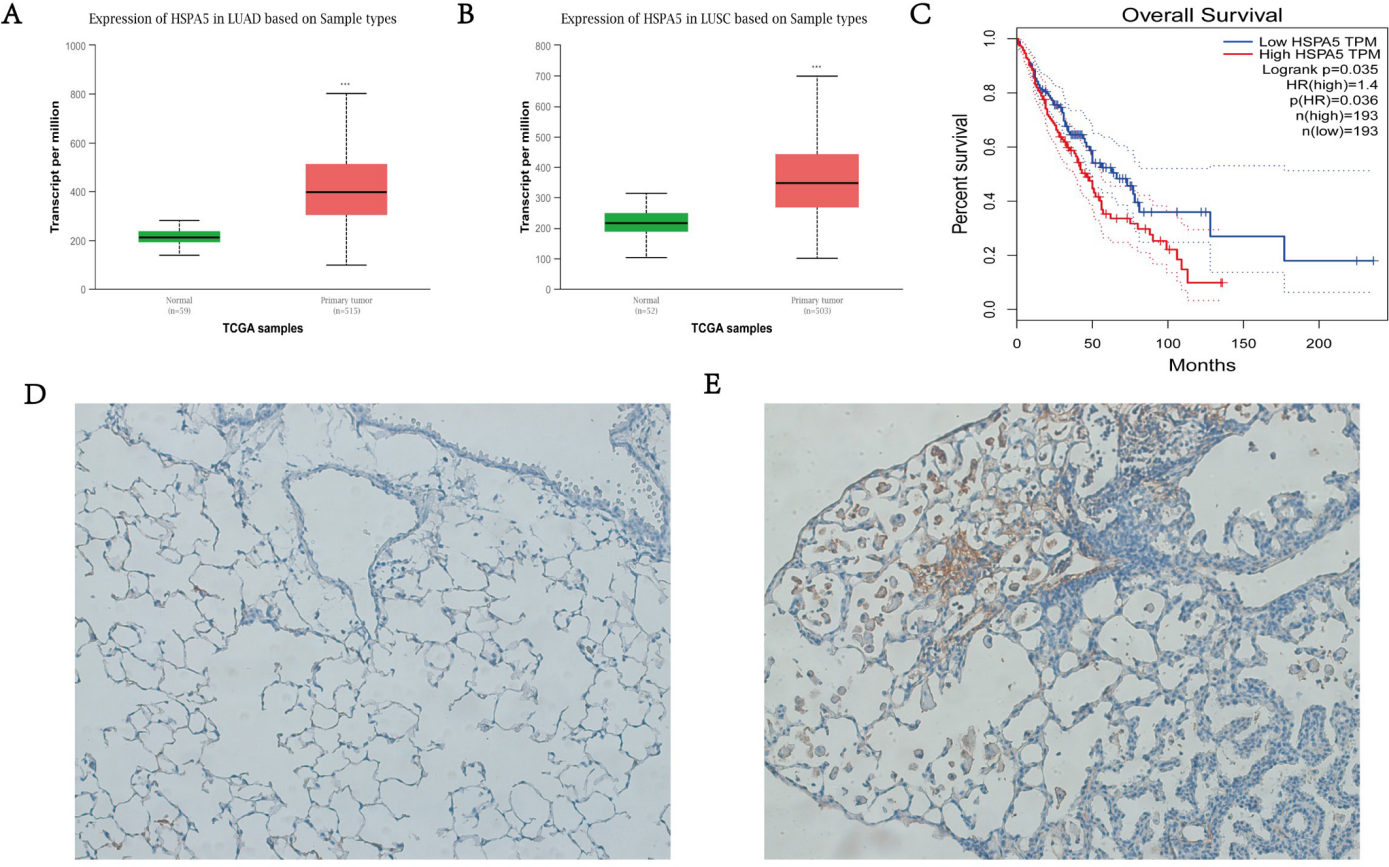
**GRP78 was highly expressed in lung cancer and related to poor prognosis.** (A) GRP78 is highly expressed in LUAD from TCGA data. (B) GRP78 is highly expressed in LUSC from TCGA data. (C) Higher level of GRP78 expression is significantly related to decreased survival. (D) GRP78 expression in lung tissue of wild-type mouse. (E) GRP78 expression in lung tissue of lung cancer mouse model. Original magnification, x 200; ***P<0.001; LUAD, Lung adenocarcinoma; LUSC, lung squamous cell carcinoma.

By analyzing the publically available database, the Cancer Genome Atlas (TCGA), and the Genotype-Tissue Expression project (GTEx), using the methods published previously (Chandrashekar et al., 2017; Tang et al., 2017), we found that GRP78 was significantly upregulated in primary lung cancer tissue compared to normal lung tissue, both in lung adenocarcinoma (Fig. 1A) and lung squamous cell carcinoma (Fig. 1B). In addition, survival analysis showed that high expression of GRP78 was indicative of a poor prognosis (Fig. 1C). The same situation was observed in mice: compared with control group (Fig. 1D), GRP78 was found to be highly expressed in the mice with lung cancer (Fig. 1E). These results suggest that GRP78 might be a biomarker in tumorigenesis, and of an independent poor prognosis.

### Targeted inhibition of GRP78 attenuates viability of lung cancer cells

**Fig. 2.**
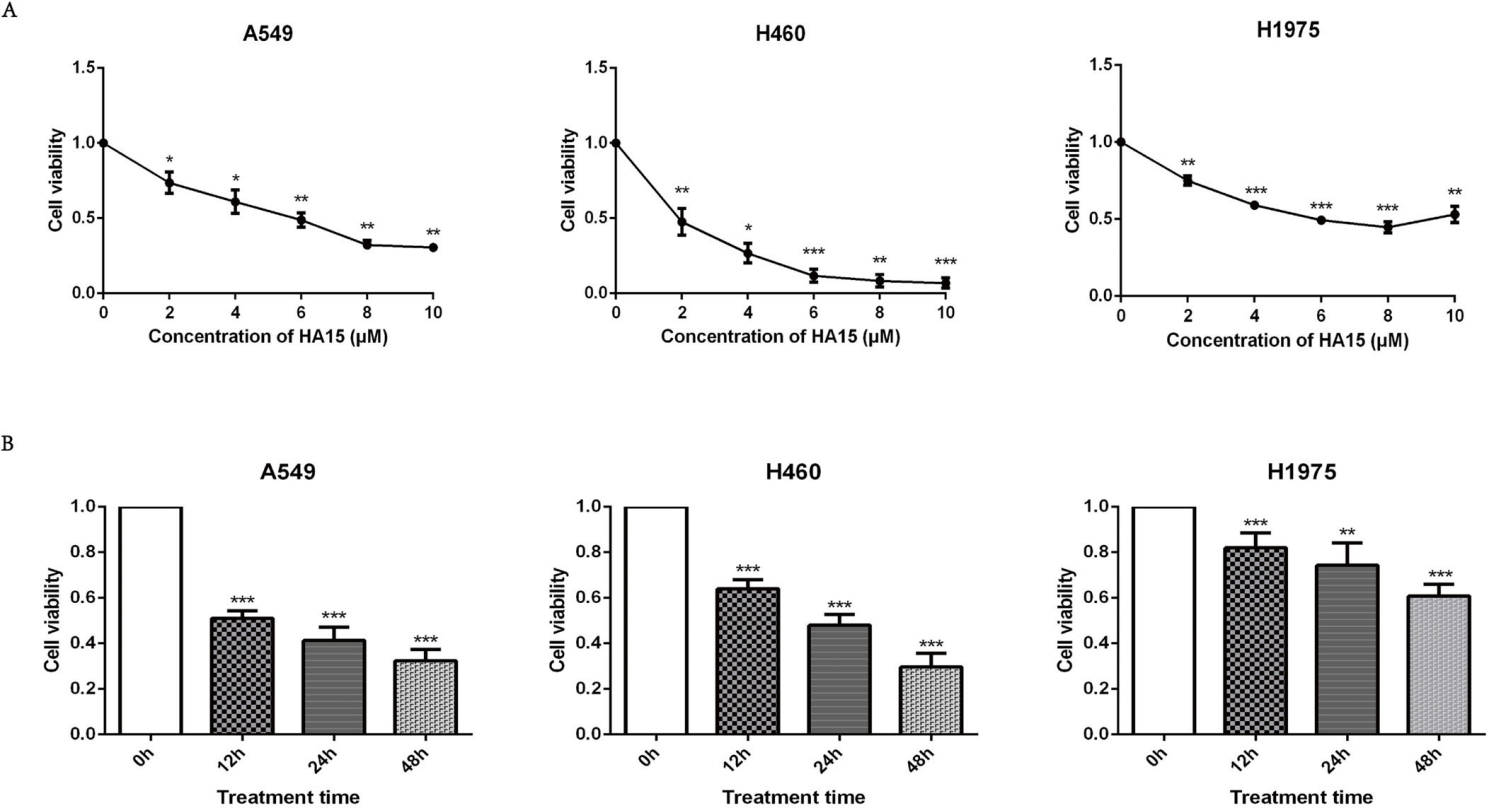
**Cell viabilities of A549, H460, and H1975 were significantly decreased in a dose-dependent and time-dependent manner after treatment with HA15.** (A) The cell viability of A549, H460, and H1975 cells after treatment with increasing concentrations of HA15 for 48 h. (B) The cell viability of A549, H460, and H1975 cells after treatment with 10 μM HA15 for increasing time. *n*=3 individual experiments, *P<0.05, **P<0.01, ***P<0.001.

The lung cancer cell lines A549, H460, and H1975 were treated with different HA15 concentrations of 0, 2, 4, 6, 8, and 10 μM. After 48 h, cell viability was determined by the CCK-8 assay. We found that, compared with the control group (0 μM), the viability of HA15-treated lung cancer cells decreased significantly with increasing concentrations of HA15 (Fig. 2A). Higher concentrations, 20, 50, and 100 μM, of HA15 treatment showed no further inhibition effect on cell viability (Fig. S1). When lung cancer cells were treated with HA15 (10 μM) for different time-periods (12 h, 24 h, and 48 h), cell viability declined significantly in a time-dependent manner (Fig. 2B).

### Targeted inhibition of GRP78 inhibits lung cancer cell proliferation and promotes apoptosis

**Fig. 3.**
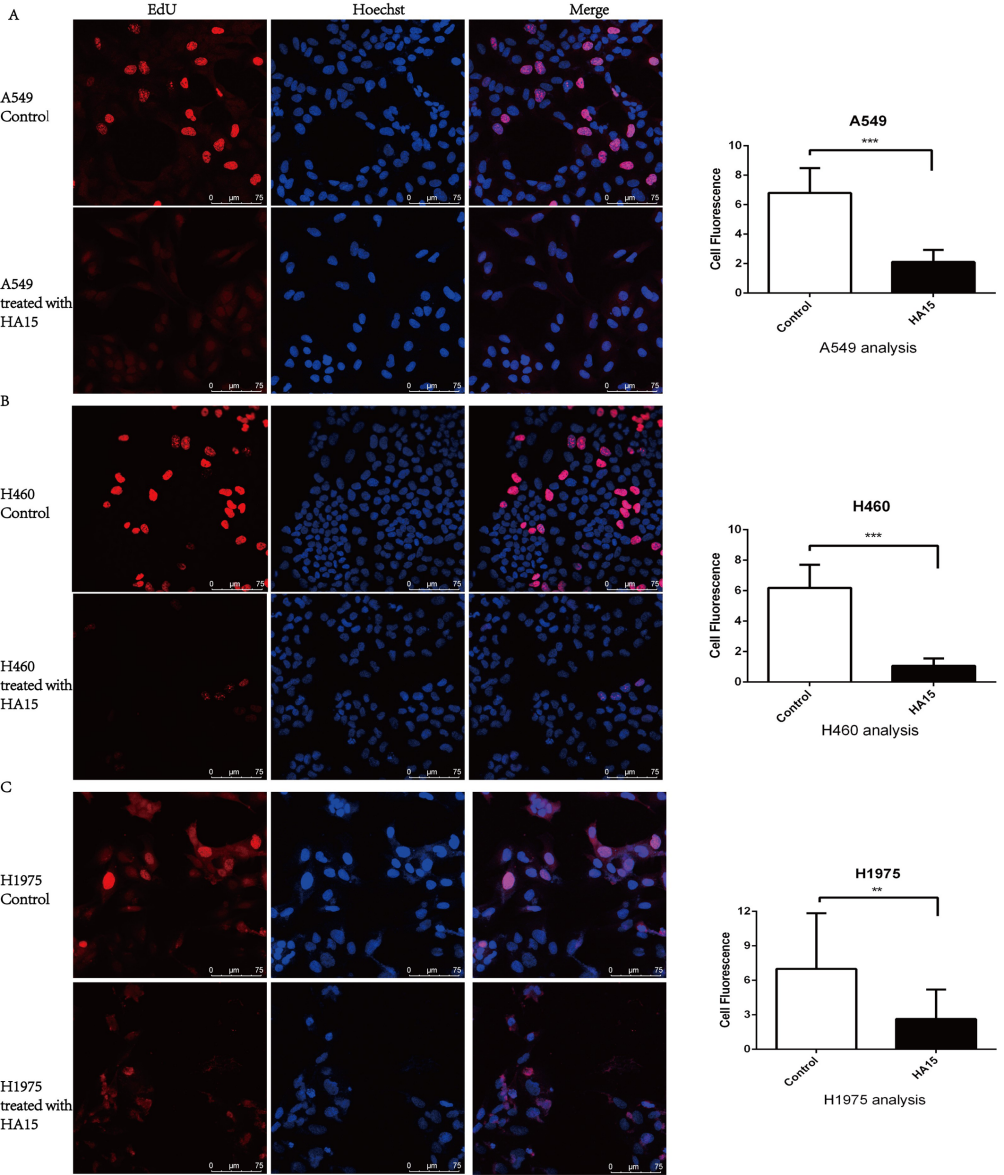
**Inhibition of GRP78 by HA15 suppressed lung cancer cells proliferation.** (A–C) The EdU assay result of A549, H460, and H1975 cells treated with 10 μM HA15 for 24 h. *n*=3 individual experiments, **P<0.01, ***P<0.001.

**Fig. 4.**
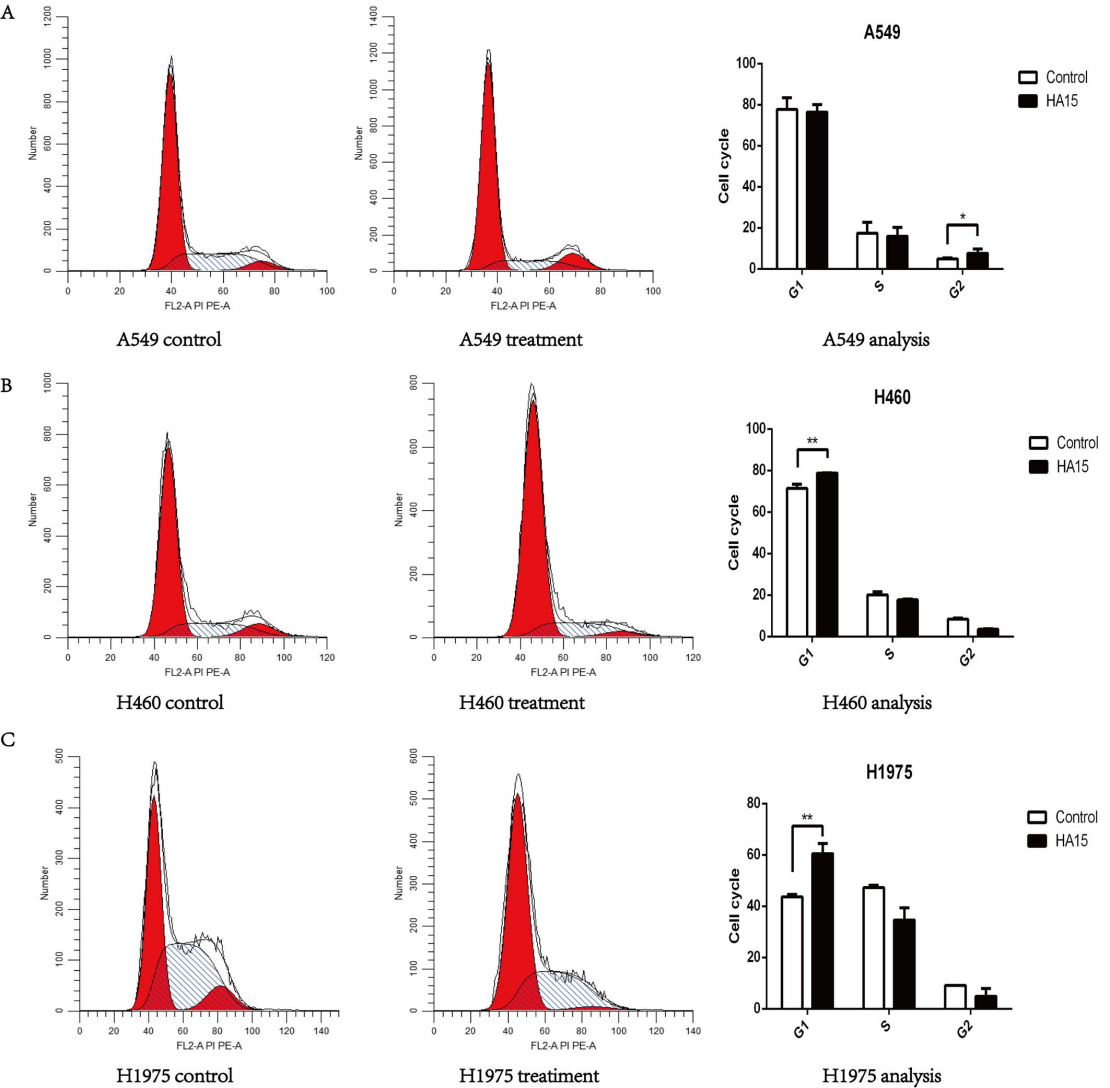
**Inhibition of GRP78 by HA15 changed cell cycles in lung cancer cells.** (A–C) The cell cycle changed of A549, H460, and H1975 cells treated with 10 μM HA15 for 24 h. *n*=3 individual experiments, *P<0.05, **P<0.01.

**Fig. 5.**
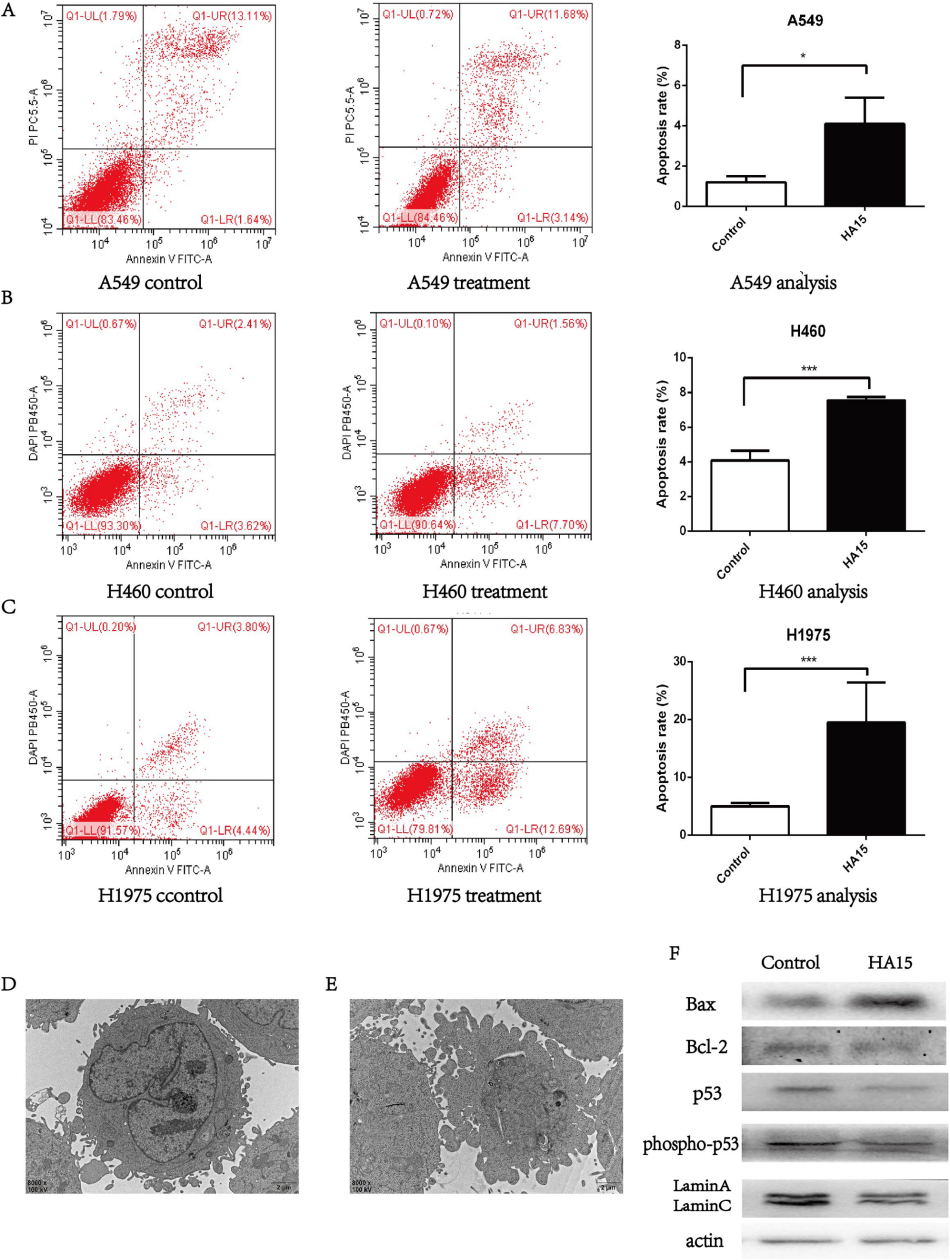
**Inhibition of GRP78 by HA15 promoted apoptosis in lung cancer cells.** (A–C) The apoptosis rate of A549, H460, and H1975 cells increased significantly after treated 10 μM HA15 for 24 h. (D,E) The representative electron microscopic picture of A549 without or with 10 μM HA15 treatment. (F) The expression of Bax, Bcl-2, p53, phospho-p53, and LaminA/C in A549 cells after treated with 10 μM HA15 for 24 h tested by western blot. *n*=3 individual experiments, *P<0.05, ***P<0.001.

To further explore the role of GRP78 in lung cancer cells, we used the EdU assay and flow cytometry to analyze the proliferation rates, cell cycle arrests and apoptosis rates of A549, H460, and H1975 cells after treatment with 10 μM HA15 for 24 h. We found that the proliferation of all lung cancer cells was inhibited by HA15 (Fig. 3). Cell cycle analysis showed that A549 cells were arrested in the G2/M phase (Fig. 4A), H460 cells and H1975 cells were blocked in the G1/G0 phase (Fig. 4B and C). In addition, the apoptosis rates of A549 (Fig. 5A), H460 (Fig. 5B), and H1975 (Fig. 5C) cells were significantly higher in HA15-treated cells than the respective control groups. Compared to the untreated group (Fig. 5D), electron microscopy images of A549 cells treated for 24 h with HA15 displayed membrane blebs characteristic of apoptotic cells (Fig. 5E). The protein expression levels of Bcl-2 and p53 activation were decreased, but Bax expression was increased after 24 h treatment with HA15. Consistently, the expression of LaminA/C were decreased (Fig. 5F).

### HA15-induced apoptosis was concomitant with ER stress

**Fig. 6.**
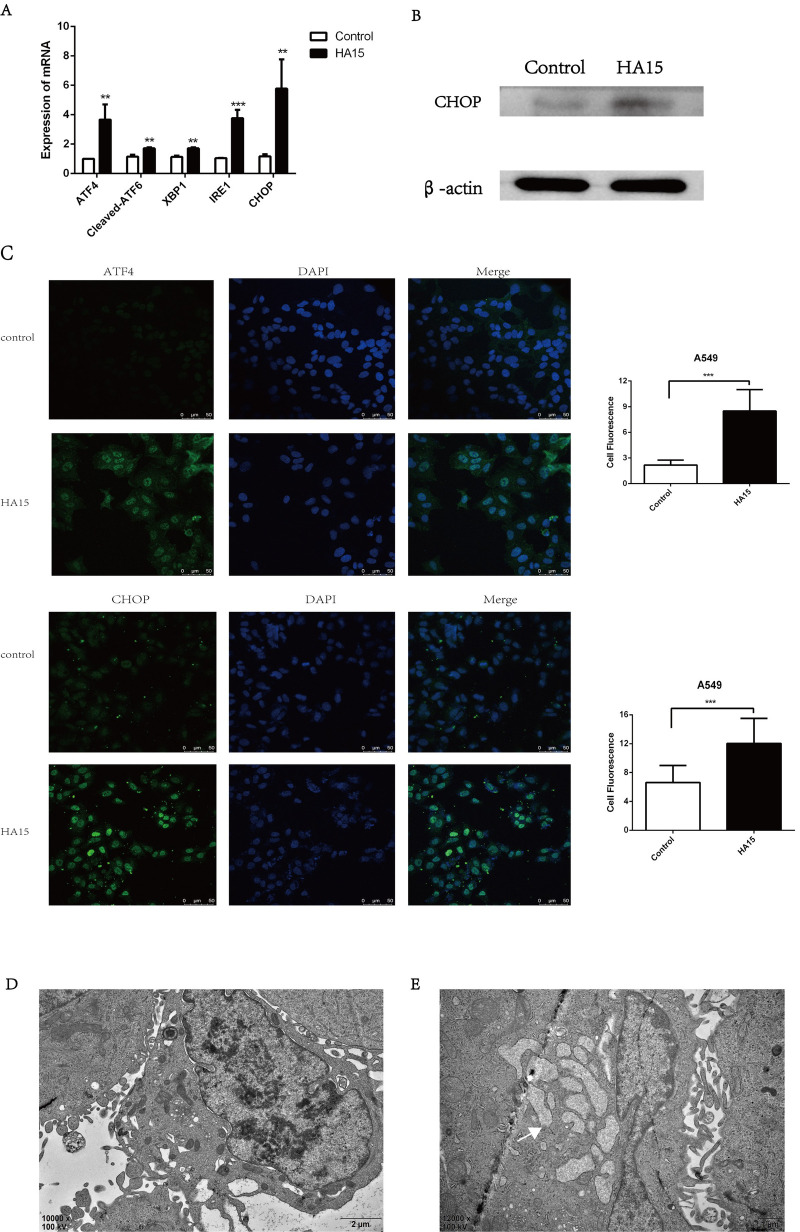
**Inhibition of GRP78 by HA15 induced ER stress.** (A) The expression of *ATF4*, *ATF6*, *XBP1*, *IRE1* and *CHOP* in A549 cells treated with 10 μM HA15 for 24 h tested by RT-PCR. (B) The expression of CHOP in A549 cells treated with 10 μM HA15 for 24 h tested by western blot. (C) The nuclear compartmentalization of ATF4 and CHOP in A549 cells treated with 10 μM HA15 for 24 h detected by immunofluorescence assay. (D,E) The representative ER electron microscopic picture in A549 cells without or with 10 μM HA15 treatment. The dilated ER is indicated by an white arrow. *n*=3 individual experiments, **P<0.01, ***P<0.001.

Since GRP78 is the pivotal chaperone protein in ER stress, we analyzed the expression of ER stress-related mRNAs and proteins. We found that the expression of *ATF4*, *ATF6*, *XBP1*, *IRE1*, and *CHOP* were significantly increased at the mRNA level; all involved in the signaling network of the unfolded protein response (Fig. 6A). The protein expression of CHOP, a pro-apoptotic factor of ER stress, was also significantly increased (Fig. 6B). In addition, immunofluorescence staining showed the increased nuclear compartmentalization of CHOP and ATF4 in response to 10 μM HA15 treatment in A549 cells (Fig. 6C). Furthermore, compared to the control group (Fig. 6D), electron microscopy revealed that the A549 cells exposed to HA15 (10 μM) displayed typically dilated ER cisternae, a classical feature of ER stress (Fig. 6E).

### HA15-induced autophagy in A549 cells

**Fig. 7.**
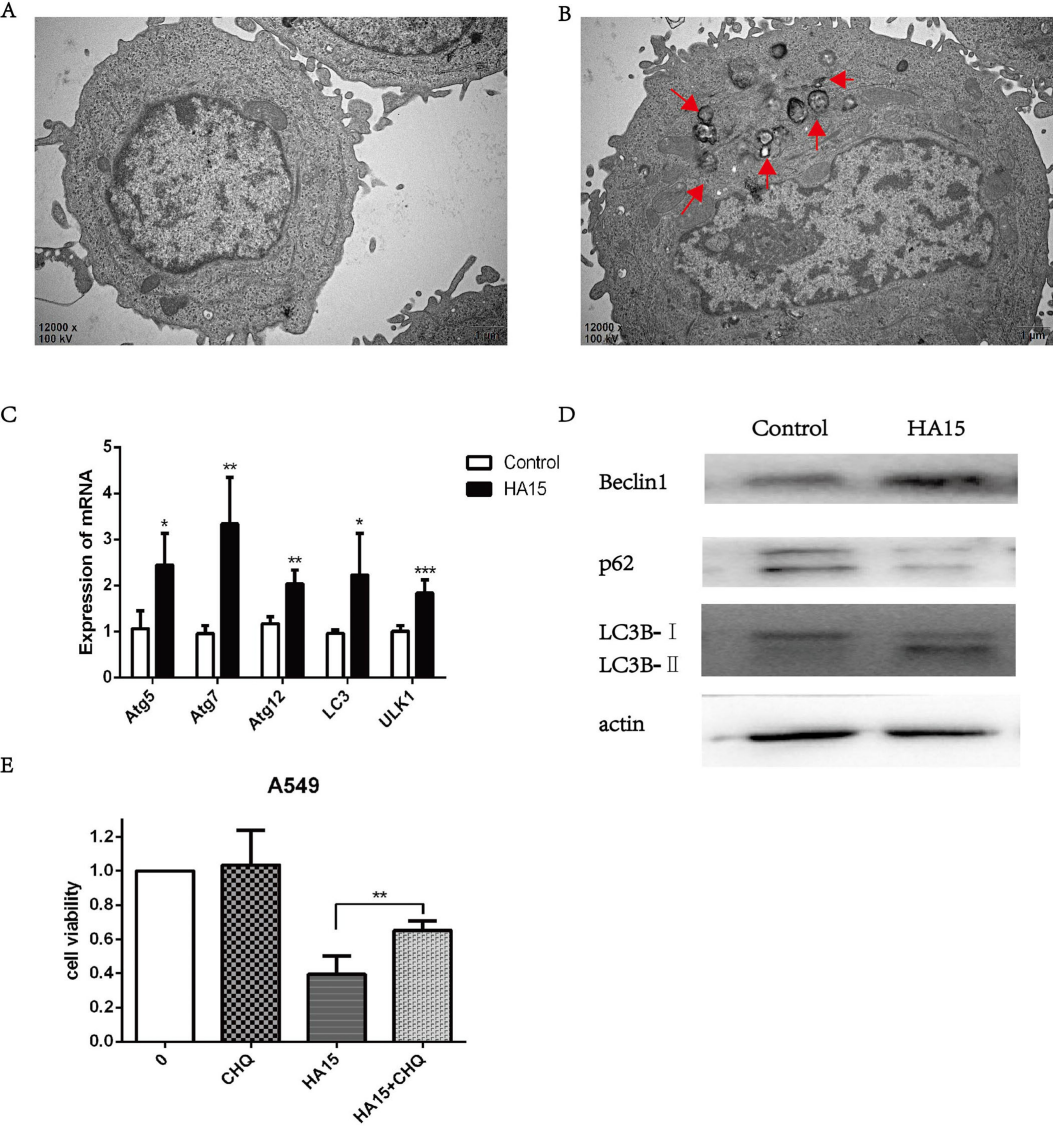
**Inhibition of GRP78 by HA15 triggered autophagy.** (A) The representative electron microscopic picture of A549 cell without HA15 treatment. (B) The representative autophagosome electron microscopic picture of A549 treated with 10 μM HA15 for 24 h. The autophagic vacuoles are indicated by red arrows. (C) The expressions of *Atg5*, *Atg7*, *Atg12*, *LC3* and *ULK1* in A549 cells treated with 10 μM HA15 for 24 h tested by RT-PCR. (D) The expressions of Beclin1, p62, LC3B in A549 cells treated with 10 μM HA15 for 24 h tested by western blot. (E) The cell viability of A549 cells after treated with 10 μM chloroquine (CHQ) for 6 h and 10 μM HA15 for 24 h. *n*=3 individual experiments, *P<0.05, **P<0.01, ***P<0.001.

Electron microscopy images of A549 cells treated with 10 μM HA15 showed an accumulation of numerous vesicles with a distinct double membrane, a typical feature of autophagy (Fig. 7B). In contrast, the control A549 cells did not show any autophagic vacuoles (Fig. 7A). These ultra-structural hallmarks of autophagy led us to investigate whether HA15 might also induce autophagy. Consistent with the results from the electron microscopy images, we found that the expression of *Atg5*, *Atg7*, *Atg12*, *LC3*, and *ULK1*, which are all related to autophagy, were significantly upregulated after treatment with 10 μM HA15 for 24 h (Fig. 7C). Furthermore, HA15 upregulated the protein expression of Beclin-1, which is essential for initiating the formation of the autophagosome in autophagy. At the same time, the protein expression of p62 was downregulated by HA15, which indirectly reflects the decreased level of autophagosome clearance. Consistent with that, decreased expression of LC3B-I and increased expression of LC3B-II were observed after HA15 treatment (Fig. 7D). To investigate further whether the upregulated autophagy lead to cell death in this condition, we used the autophagy inhibitor chloroquine (CHQ) to treat A549 cells together with HA15. The results showed that the cell viability of HA15 combined with CHQ was restored compared with the HA15 treatment group (Fig. 7E).

## DISCUSSION

GRP78, the key protein in the ER, is implicated in various diseases, such as tumors and neurodegenerative diseases (Hebert-Schuster et al., 2018; Casas, 2017). In the current study, we found that GRP78 was highly expressed in lung adenocarcinoma and lung squamous cell carcinoma, and was associated with a poor prognosis. Furthermore, inhibition of GRP78 by HA15 significantly decreased the viability of A549, H460, and H1975 cells in a dose- and time-dependent manner. HA15 inhibited A549, H460, and H1975 cell proliferation and promoted apoptosis. ER stress and autophagy were triggered by HA15 in A549 cells and these processes were involved in HA15-induced apoptosis.

It has been reported that high expression of GRP78 could promote tumorigenesis and drug resistance, and inhibition of GRP78 could improve the efficacy of chemotherapy drugs (Huang et al., 2016; Dauer et al., 2018; Cook and Clarke, 2015). A clinical study, including 163 peripheral blood samples from non-small-cell lung cancer patients, revealed that GRP78 is highly enriched in advanced stages, significantly higher than seen in early-stage patients, which may be important in the carcinogenesis of non-small-cell lung cancer, and is associated with a poor prognosis (Ma et al., 2015). Our bioinformatic analysis showed that GRP78 was also higher in patients with lung cancer than in healthy people at the transcriptomic level, indicating that GRP78 may be associated with lung tumorigenesis and development.

It has been reported that HA15, a novel inhibitor targeting GRP78, could upregulate ER stress levels in malignant pleural mesothelioma cells, induce the pro-apoptotic UPR and autophagy, and induce cell death (Xu et al., 2019). Similarly, lung cancer cell apoptosis induced by HA15 was concomitant with ER stress. In this study, we found that after HA15 treatment, the pro-apoptotic UPR signaling was activated, resulting in significantly increased expression of CHOP. Electron microscopy images of A549 cells exposed to HA15 displayed typical dilated ER cisternae, which is a classical feature of ER stress, indicating that ER stress is also involved in HA15-induced apoptosis in lung cancer cells. It seems strange that HA15 can inhibit GRP78 and trigger ER stress at the same time. Cerezo et al., (2016) demonstrated that while HA15 induced dissociation of the GRP78, protein kinase RNA (PER)-like ER kinase (PERK), IRE1α, and ATF6 complex, thus inducing ER stress, GRP78 binds ATP with high affinity and its ATPase activity, stimulated by binding to the unfolded protein, catalyzes re-folding. HA15 is able to inhibit the ATPase activity of GRP78 in a dose-dependent manner, leading to the accumulation of unfolded proteins and aggravating ER stress.

HA15-induced apoptosis is not only related closely to ER stress but also to autophagy (Cerezo et al., 2016; Xu et al., 2019). Analogously, we found that HA15 treatment of the lung cancer cell line, A549, activated autophagy and triggered formation of autophagosomes. Since autophagy is considered a double-edged sword relating to cell death, we used chloroquine to block the autophagy process, and found that the cell viability of HA15 together with CHQ treatment was restored compared with the HA15 treatment alone, which elucidated that upregulated autophagy in this condition promoted the programmed cell death. It has been reported that the transcription factors ATF4 and CHOP could increase the transcription of the autophagy gene *MAP1LC3B*, which encodes LC3B, and ATG5, phagocytic expansion and autophagosome formation (Rouschop et al., 2010). Interestingly, we found that ATF4 and CHOP were upregulated after treatment with HA15, which may increase autophagy. The *ATG* genes controlling the formation of autophagosomes through Atg12-Atg5 and LC3B complexes, were upregulated after HA15 treatment.

In summary, GRP78 is highly expressed in patients with lung cancer and is associated with a poor prognosis. Targeted inhibition of GRP78 by HA15 promoted lung cancer cells’ apoptosis, through involvement of ER stress and autophagy. Targeting the inhibition of GRP78 with HA15 may become a new target for clinical treatment and drug development in the future.

## MATERIALS AND METHODS

### Animals and experimental design

The protocols for both laboratory animal handling, and experiments, were in strict accordance with the Chongqing Management Approach of Laboratory Animals (Chongqing government order number 195). Mouse breeding and experimental procedures were approved by the Animal Medical Committee of Chongqing Medical University (Chongqing, China). Animal surgery was performed under sodium pentobarbital anaesthesia, and all efforts were made to minimize animal suffering.

Healthy, 6- to 8-week-old male wild-type C57BL/6J mice were purchased from the Experimental Animal Center of Chongqing Medical University and housed in a specific, pathogen-free animal laboratory (with constant temperature and humidity, 12 h light/dark cycles, adequate sterile food and drinking water). A urethane-induced C57BL/6J mouse lung cancer model was established according to our previously published protocol (Chen et al., 2017). Briefly, all mice in the experimental group received a weekly intraperitoneal injection of urethane (1 mg/***g*** body weight, Sigma-Aldrich, USA), and the control group receiving the same volume of saline, for 10 consecutive weeks. Mice were sacrificed after a 15 week latency period.

### Immunohistochemistry assay

After the mice were anesthetized, the lungs were removed and fixed in paraformaldehyde for 24 h, before dehydration with gradient alcohol. Subsequently they were embedded in paraffin, and sectioned at a thickness of 5μm for immunohistochemistry assays. Sections were incubated with antibodies against GRP78 (1:2000, a kind gift from Professor Deqiang Wang, Chongqing Medical University) overnight at 4°C. The corresponding secondary antibody was added to the slice and incubated at 37°C for 30 min. A 3,3′-diaminobenzidine (DAB) Kit (ZSGB-BIO, Beijing, China) was then used as a chromogenic agent. Slices were put into hematoxylin solution for counterstaining, then dehydrated and sealed, before the protein expression in tissues was observed under a microscope.

### Cell culture

The adenocarcinoma human alveolar basal epithelial cell line A549 was purchased from the American Type Culture Collection (ATCC), and both the human large cell lung cancer cell line NCI-H460 (H460) and the human non-small cell lung cancer cell line NCI-H1975 (H1975) were purchased from Stem Cell Bank, Chinese Academy of Sciences (Shanghai, China). All cell lines were authenticated by Microread Genetics Co., Beijing, China. The A549, H460 and H1975 cells were maintained in Roswell Park RPMI-1640 media (Biological Industries, Israel), supplemented with 10% fetal bovine serum (Biological Industries, Israel) and 100 U/ml penicillin and streptomycin (HyClone, USA) at 37°C in a 5% CO_2_ incubator.

### Cell viability assays

Cell viability was assessed using the Cell Counting Kit (CCK)-8 assay (MCE, USA). A549, H460 and H1975 cells were plated in 96-well plates at appropriate cell densities and cultured for 24 h in RPMI-1640 growth media as described above. After a subsequent 12 h starvation in serum-free media (RPMI-1640 media without fetal bovine serum), lung cancer cells were treated with GRP78 targeting inhibitor, HA15 (MCE, USA), at increasing concentrations (0–100 μM). At the indicated time point, 10 μL of CCK-8 solution was added to the wells, and the absorbance of each well was measured to calculate the cell viability (%).

To check the effect of autophagy on cell viability, autophagy inhibitor chloroquine (CHQ, MCE, USA) was used to treat A549 cells together with HA15. The cells were pretreated with 10 μM chloroquine for 6 h, and then 10 μM HA15 was added for 24 h. Cell viability was determined by the CCK-8 assay.

### Cell proliferation assays

Cell proliferation was assessed using the EdU (Beyotime, Shanghai, China) assay. A549, H460 and H1975 cells were plated in six-well plates with slides placed and cultured for 24 h in RPMI-1640 media. After treatment with 10 μM HA15 for 24 h, cells were incubated with 10 μM EdU working solution (5-ethynyl-2′-deoxyuridine, preheated to 37°C) for 2 h. The cells were fixed with 4% paraformaldehyde and permeabilized with 0.3% Triton X-100. Next, 0.5 mL Click reaction solution was added to each slide, and nuclei were stained with Hoechst. Imagines were obtained with an inverted fluorescence microscope.

### Flow-cytometric analysis

Cell cycle changes and apoptosis were detected using cell flow cytometry. The untreated A549, H460 and H1975 cells, and cells treated with 10 μM HA15 for 24 h, were rinsed twice with PBS. The cells were then digested with trypsin, collected, and centrifuged at 1000 rpm for 3 min. Cells intended for cell cycles analysis were fixed with 1 mL ethanol for 24 h at 4°C, washed with PBS, and incubated with 1 mL propidium iodide (PI) for 30 min in the dark. DNA content was measured based on the presence of PI-stained cells. The cells used for the detection of apoptosis were resuspended in 1 mL PBS and analyzed using flow cytometry after staining with Annexin V and DAPI.

### Western blot analysis

A549 cells were incubated with 10 μM HA15 for 48 h. The cells were then harvested and whole proteins extracted with 100 μL RIPA buffer containing 5 μL protease inhibitor (20×), 2 μL phosphatase inhibitor, and 1 μL 100 mM phenylmethanesulfonyl fluoride. Proteins were extracted using sodium dodecyl sulfate-polyacrylamide gel electrophoresis (Bio-Rad, CA, USA). The primary antibodies used in the study were: GRP78 (1:10,000, a kind gift from Professor Deqiang Wang, Chongqing Medical University), B-cell lymphoma 2 (Bcl-2)-associated X protein (Bax) and Beclin-1 (50599 and 11306, both 1:1000, Protein tech, Wuhan, China), Bcl-2 and p53 (BM0200 and BM0101, both 1:200, BOSTER, China), p62 (WL02385, 1:1000, Wanleibio, China), and actin (TA-09, 1:1000, ZSGB-BIO, China), LC3B (A5202, 1:500,bimake,USA), LaminA/C (4777, 1:2000, Cell Signaling Technology, MA, USA), Phospho-p53(ser15) (9284, 1:500, Cell Signaling Technology, MA, USA).

### Immunofluorescence assay

The nuclear compartmentalization of ATF4 and CHOP was detected using immunofluorescence. After treating with 10 μM HA15 for 24 h, cells were fixed with 4% paraformaldehyde, then stained with a primary antibody ATF4 (60035, 1:200, Protein tech, Wuhan, China) or CHOP (15204, 1:200, Protein tech, Wuhan, China) overnight at 4°C. The next day, a FITC-tagged secondary antibody (ZF0311 or ZF0312, 1:1000, ZSGB-BIO, Beijing, China) and DAPI (C1002, Beyotime, Shanghai, China), were added. All images were captured using a Leica Application Suite X (LAS X) system on a Leica microscope (Leica TCS SP8, Wetzlar, Germany).

### RNA extraction and quantitative PCR

The total RNA of cells was extracted with Trizol (Takara, Dalian, China). cDNA was amplified using PrimeScript™ RT reagent Kit with gDNA Eraser (Takara, Dalian, China). Real-time PCR was carried out in a 10 μL reaction volume containing 5 μL TB Green™ Premix Ex Taq™ II (Takara, Dalian, China), 0.5 μL each forward and reverse primers (10 μM), 1 μL cDNA and 3 μL ddH2O. A CFX connect Real-time System (Bio-Rad, CA, USA) was used for amplification with 1 cycle of 95°C for 30 s; 34 cycle of 95°C for 5 s and 56°C for 45 s. Fluorescence signals were collected after each amplification step.

The relative expression of genes was calculated by the ΔΔCt [2(^-ΔΔCt)] method, in which Ct represents the threshold cycle. The forward and reverse primers used were as follows: β-actin, 5′-CCTGGCACCCAGCACAAT-3′ and 5′-GCCGATCCACACGGAGTA-3′; Activating transcription factor (ATF) 4 (*ATF4*), 5′-GACCGAAATGAGCTTCCTGA-3′ and 5′-ACCCATGAGGTTTGAAGTGC-3′; *Cleaved ATF6*, 5′-GCTTCCAGCAGCACCCAAGAC-3′ and 5′-CGTCTGGCCTTTAGTGGGTGCA-3′; X-box-binding protein 1 (*XBP1*), 5′-CCCTCCAGAACATCTCCCCAT-3′ and 5′-ACATGACTGGGTCCAAGTTGT-3′; Inositol-Requiring Enzyme 1 (*IRE1*), 5′-GAAGACGTCATTGCACGTGAATT-3′ and 5′-AGGTCCTGAATTTACGCAGGT-3′; CCAAT-enhancer binding protein homologous protein (*CHOP*), 5′-GAACGGCTCAAGCAGGAAATC-3′ and 5′-TTCACCATTCGGTCAATCAGAG-3′; Autophagy-related protein (Atg) 5 (*Atg5*), 5′-TCAGAAGCTGTTTCGTCCTGT-3′ and 5′-TTTCCAACATTGGCTCAATTC-3′; *Atg7*, 5′-ATGATCCCTGTAACTTAGCCCA-3′ and 5′-CACGGAAGCAAACAACTTCAAC-3′; *Atg12*, 5′-TAGAGCGAACACGAACCATCC-3′ and 5′-CACTGCCAAAACACTCATAGAGA-3′; microtubule-associated protein 1A/1B-light chain 3 (*LC3*), 5′-CGATACAAGGGTGAGAAGCAG-3′ and 5′-ACACCTCTGAGATTGGTGTGG-3′; and unc-51-like kinase 1 (*ULK1*), 5′-CCAGAGCAACATGATGGCG-3′ and 5′-CCTTCCCGTCGTAGTGCTG-3′.

### Electron microscopy

The untreated A549 cells, and cells treated with 10 μM HA15, were incubated for 24 h. The cells were then digested with trypsin, collected, and centrifuged at 800 rpm for 5 min, forming loose clumps at the bottom of the tube. Most of the supernatant was then aspirated, leaving approximately 1.5 mL remaining, which was used to resuspend the cells by pipetting. The suspension was then transferred to a 2 mL Eppendorf tube, and centrifuged at 1200 rpm for 10 min, causing the cells to form a tight clump at the bottom of the Eppendorf tube. The supernatant was removed and glutaraldehyde was added for fixation. After three washes in the PBS (0.1 M), for a total of 30 min, cells were fixed with 1% v/v osmium tetroxide in 35°C for 1 h. Then cells were dehydrated in a graded ethanol series (50–100%) for 10 min each, followed by treatment with propylene oxide for 10 min. Next, the mixture of EPON 812 and propylene oxide was used to infiltrate cells for 30 min. Last, cells were embedded in EPON 812 and left to polymerize at 60°C for 1 days. Slice thickness was 70 nm, and uranium acetate and lead citrate were used for double staining. Cell sections were observed under JEOL JEM-1400plus electron microscope (Jeol, Tokyo, Japan).

### Statistical analysis

All data were obtained from three individual experiments. The values in this study are expressed as mean±standard deviation. Differences between groups were compared using the Student's *t*-test or one-way analysis of ANOVA with LSD post hoc tests. Statistical significance was set at *P*<0.05. Statistical analysis was performed using the statistical package for the Social Sciences Software Version 11.0 (SPSS, IL, USA).

## Supplementary Material

Supplementary information
