# Supplementary material for: Targeted inhibition of GRP78 by HA15 promotes apoptosis of lung cancer cells accompanied by ER stress and autophagy
Source: Biol Open. 2020 Nov 12;9(11):bio053298. doi: 10.1242/bio.053298 (PMC7673357; doi:10.1242/bio.053298)
Supplement: Supplementary information [file biolopen-9-053298-s1.pdf]

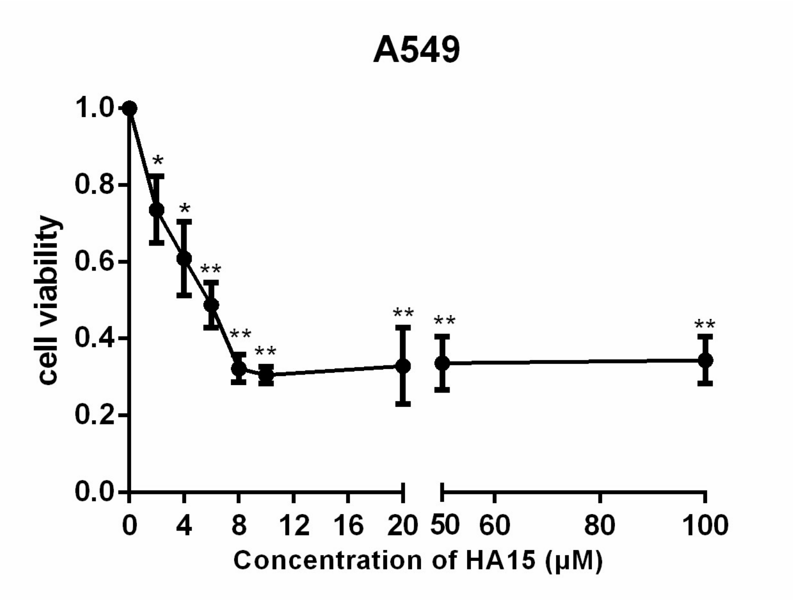

Figure S1 The cell viability of A549 was significantly decreased in a dose-dependent manner after treated with HA15 for 48h. The cell viability of A549 cells after treated with increasing concentrations of HA15 for 48h. n=3 individual experiments, *$P<0.05$, **$P<0.01$
